# Nanodevice Approaches for Detecting Micro- and Nanoplastics in Complex Matrices

**DOI:** 10.3390/nano16010055

**Published:** 2025-12-31

**Authors:** Rita Paola Debri, Fabrizia Sepe, Silvia Romano, Nicolantonio D’Orazio, Antonino De Lorenzo, Anna Calarco, Raffaele Conte, Gianfranco Peluso

**Affiliations:** 1 Faculty of Medicine and Surgery, Saint Camillus International University of Health Sciences, Via di Sant’Alessandro 8, 00131 Rome, Italy; rpaola.debri@unicamillus.org (R.P.D.); gianfranco.peluso@unicamillus.org (G.P.); 2 Research Institute on Terrestrial Ecosystems (IRET)-CNR, Via Pietro Castellino 111, 80131 Naples, Italy; fabriziasepe@cnr.it (F.S.); anna.calarco@cnr.it (A.C.); 3 Department of Experimental Medicine, University of Campania “Luigi Vanvitelli”, Via Santa Maria di Costantinopoli 16, 80138 Naples, Italy; silvia.romano@iret.cnr.it; 4 Department of Medical, Oral and Biotechnological Sciences, University “G. D’Annunzio”, Via dei Vestini, 31, 66100 Chieti, Italy; n.dorazio@unich.it; 5 Department of Biomedicine and Prevention, University of Rome “Tor Vergata”, Via Montpellier 1, 00133 Rome, Italy; delorenzo@uniroma2.it; 6 National Biodiversity Future Center (NBFC), 90133 Palermo, Italy

**Keywords:** microplastics, nanoplastics, nanodevices, environmental monitoring, biosensors, lab-on-a-chip, microfluidics, nanopore

## Abstract

Micro- and nanoplastics (MNPs) are increasingly recognized as pervasive environmental contaminants with profound implications for ecosystems and human health. Their small size, compositional diversity, and occurrence across complex matrices—including water, soil, food, and biological samples—pose substantial analytical challenges. Conventional techniques such as vibrational spectroscopy, chromatographic analysis, and electron microscopy have yielded critical insights into MNP composition, morphology, and distribution; however, these methods often face limitations in sensitivity, throughput, and adaptability to real-world samples. Recent advances in nanotechnology have catalyzed the emergence of nanodevices—encompassing nanosensors, nanopore systems, integrated lab-on-a-chip platforms and nanostructured capture materials—that promise enhanced sensitivity, specificity, and the capacity for real-time, in situ detection. These innovations not only facilitate high-throughput analysis but also provide novel opportunities for integrated characterization of MNPs across diverse matrices. This review synthesizes the current state of nanodevice-based MNP detection, critically examining their principles, performance, and limitations relative to conventional approaches, and outlining the key needs for standardization, matrix-specific adaptation, and regulatory harmonization.

## 1. Introduction

The proliferation of plastics in the modern environment has created a challenge of unprecedented scale. While plastics have long been valued for their durability, versatility, and low production costs, these same properties have rendered them persistent pollutants, accumulating across ecosystems and entering the food chain [1]. Microplastics, typically defined as particles smaller than five millimeters, and nanoplastics, which extend into the sub-micron scale, have garnered particular attention due to their widespread presence, high surface-area-to-volume ratio, and potential for biological interactions [2]. These particles are not confined to remote marine environments but are now documented in rivers, soils, agricultural products, processed foods, and even human tissues, raising questions about their ecological impact, bioaccumulation, and potential health consequences [2]. Detecting and characterizing micro- and nanoplastics, however, is an analytical challenge. Their small size, chemical diversity, and heterogeneous morphology complicate conventional measurement approaches [3]. Traditional techniques, including Fourier-transform infrared spectroscopy (FTIR), Raman microscopy, pyrolysis-gas chromatography/mass spectrometry (Py-GC/MS), and electron microscopy, have provided critical insights into polymer identity, size distribution, and abundance. These methods, however, require extensive sample preparation, are time-intensive, and suffer of low sensitivity when analyzing nanoplastics or complex matrices [3]. Moreover, environmental and biological samples are heterogeneous and characterized by the presence of organic matter, minerals, and other particulates capable of interfering with analytical signals, further limiting the effectiveness of conventional approaches [3]. Nanotechnology has emerged as a transformative avenue for advancing MNP detection. Nanodevices leverage the unique properties of nanomaterials, including high surface area, tunable chemical reactivity, and exceptional sensitivity, to overcome many of the problems of traditional methods [4]. Electrochemical sensors, optical nanosensors, nanopore systems, and lab-on-a-chip platforms have demonstrated the capacity for rapid, sensitive, and, in some cases, real-time detection of micro- and nanoplastics in diverse environments [5]. These devices can integrate sample pre-treatment, separation, and detection within a single compact platform, offering the potential for high-throughput monitoring and in situ analysis in settings ranging from remote field sites to industrial facilities [5]. This review aims to provide a comprehensive overview of the current state of nanodevice-based MNP detection. It examines the principles of nanotechnology-enabled platforms, explores their applications across water, soil, food, and biological matrices, and evaluates their performance relative to conventional analytical techniques.

## 2. Conventional Analytical Methods for MNP Detection

The detection and characterization of micro- and nanoplastics relies on techniques adapted from polymer science and materials characterization. These methods include spectroscopy, microscopy, thermal analysis, and mass spectrometry [6]. However, microplastics (MPs, 1 µm–5 mm) and true nanoplastics (NPs, <100 nm) differ significantly in their physicochemical behavior and detection challenges. While MPs are large enough to be detected by conventional optical or spectroscopic techniques, NPs exhibit high diffusivity, increased surface-area-to-volume ratio, and a greater tendency to aggregate or adsorb to matrix components. These characteristics make NPs much more difficult to isolate and detect, often resulting in signal attenuation or loss in standard analytical platforms. Consequently, nanoplastic detection requires higher-sensitivity techniques and tailored sample preparation strategies, whereas MPs can be monitored with relatively established methods. Recognizing these differences is critical for developing accurate, size-specific detection and quantification approaches. Moreover, significant constraints are present when these techniques are applied to complex samples or to particles at the micro-nanoscale.

### 2.1. Spectroscopic Methods

Spectroscopy has become a key tool in MNP research due to its ability to provide direct chemical fingerprints of polymers in a non-destructive manner. Among these, Fourier-transform infrared (FTIR) spectroscopy is the most widely employed [7]. FTIR identifies plastics by detecting the absorption of infrared light corresponding to characteristic functional groups. With the development of micro-FTIR imaging systems, it has become possible to automate the mapping and identification of particles down to around 10–20 μm in size [8]. This technique has proven particularly useful in studies of aquatic samples and sediments, as well as in the analysis of soil and even biological tissues. However, nanoplastics, especially those below one micrometer, remain invisible to this method. Moreover, overlapping spectra among different polymers often complicate discrimination, while organic matter in environmental matrices can interfere with the spectral signal, necessitating sample pre-treatment that may introduce bias. The process is also relatively slow when applied to large numbers of particles, making high-throughput analysis difficult [8]. Raman spectroscopy provides a complementary approach, relying on inelastic scattering of laser light to generate vibrational spectra. Compared to FTIR, Raman offers higher spatial resolution and is capable of detecting particles as small as one micrometer [9]. This advantage has made Raman particularly valuable in studies of drinking water, seafood, and atmospheric microplastic fallout. In addition, when combined with confocal microscopy, Raman spectroscopy can visualize three-dimensional distributions of particles within biological tissues, thereby providing unique insights into particle internalization and tissue penetration [10]. However, fluorescence interference from organic matter often obscures Raman signals, reducing its applicability in environmental matrices with complex backgrounds, and spectral libraries for polymer identification are still not standardized across laboratories [11]. Near-infrared (NIR) spectroscopy and hyperspectral imaging have also been applied to micro-nano plastic analysis. These techniques are particularly well-suited for large-scale monitoring and sorting applications, as they enable rapid screening of larger microplastics, typically above 100 μm [12]. However, their resolution is insufficient for small particles and their accuracy diminishes in complex environmental samples [12].

### 2.2. Microscopy-Based Approaches

Microscopy is a technique used for MNP research since it supplies important information on morphology and size distribution [13]. Optical microscopy is commonly used for initial screening of microplastics larger than 50 μm. When combined with staining agents such as Nile Red, optical microscopy enables rapid visualization of microplastics in complex matrices [14]. However, staining can lead to false positives, while small nanoplastics are outside the detection limits of conventional light microscopy [14].

For higher-resolution imaging, electron microscopy has been extensively employed. Scanning electron microscopy (SEM) provides detailed surface topography and reveals morphological features such as cracks, pitting, and erosion that are indicative of environmental weathering [15]. SEM can be coupled with energy-dispersive X-ray spectroscopy (EDS), SEM can also supply elemental composition data, which is useful in distinguishing plastics from inorganic particles [16]. However, SEM does not provide direct information on polymer identity, and the requirement for conductive coatings such as gold sputtering can alter surface features [16]. Transmission electron microscopy (TEM) offers higher resolution, capable of imaging structures at the nanometer scale. TEM has been particularly useful in visualizing nanoplastics and their interactions with cells and tissues in laboratory studies. This technique is labor-intensive, requires extensive sample preparation, and is not practical for routine monitoring in environmental contexts [17]. Atomic force microscopy (AFM) measures the mechanical properties of MNP such as elasticity and surface roughness, providing valuable insights into polymer degradation. The disadvantages related to AFM are the slow scanning speed, limited throughput, and the need for flat, stable substrates, all of which restrict its broader application in environmental monitoring [18].

### 2.3. Thermal and Mass Spectrometry-Based Techniques

**Figure 1:**
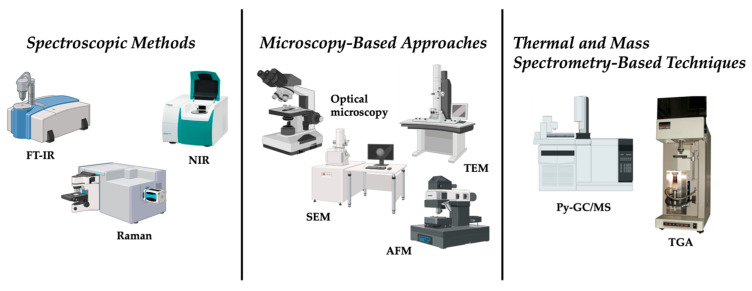
Conventional methods for micro and nano plastics detection.

Thermal degradation and mass spectrometry-based techniques represent another important category of methods, primarily used for chemical identification and quantification of polymer mass. Among these, pyrolysis gas chromatography coupled to mass spectrometry (Py-GC/MS) is the most established. In this technique, samples are thermally decomposed under inert conditions, producing volatile degradation products that are separated chromatographically and then identified via mass spectrometry [19]. Each polymer type yields a characteristic pyrolytic fingerprint, enabling precise identification and quantification [19]. Py-GC/MS has proven invaluable for analyzing sediments, sewage sludge, and biological tissues where particle isolation is difficult [20]. However, as a destructive method, it eliminates any possibility of morphological analysis, providing no information about particle size or shape [20]. Thermogravimetric analysis (TGA) measures mass loss as a MNP sample is heated. Different polymers decompose at characteristic temperatures, and when coupled with FTIR or MS for evolved gas analysis, TGA can yield additional insights into polymer composition [21]. However, the overlap in decomposition temperatures between polymers reduces specificity, particularly in mixed samples [21]. Figure 1 summarizes the conventional analytical methods for micro and nano plastics detection.

While conventional techniques are primarily laboratory-bound and used for accurate characterization, nanodevices offer enhanced sensitivity, portability, and potential for integration with automated sampling and environmental monitoring systems.

## 3. Nanodevices in MNP Monitoring

### 3.1. The Emergency of Nanodevices

The recognition that conventional approaches cannot adequately detect and characterize micro- and nanoplastics leads to the search for alternative technologies capable of addressing these analytical gaps. Among the most promising innovations is the development of nanodevices, broadly defined as analytical tools and platforms that harness the unique physicochemical properties of nanomaterials or exploit miniaturized architectures at the micro- and nanoscale [22].

**Figure 2:**
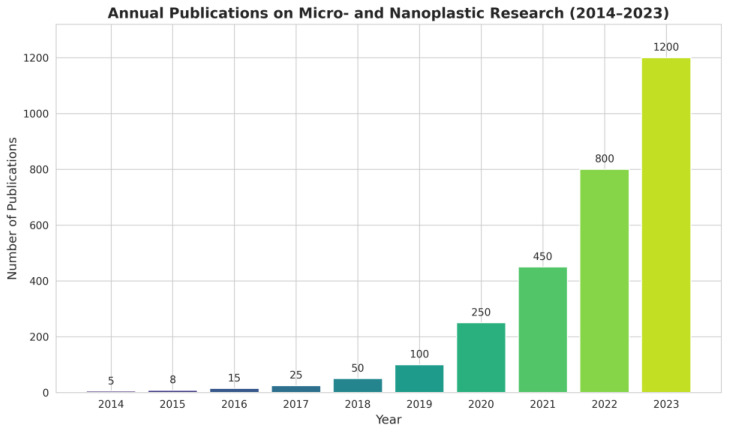
Annual trend of publications on micro- and nanoplastic research (2014–2023).

Nanodevices are particularly well suited to the challenges posed by MNP for different reasons. First, their sensitivity often surpasses that of traditional instruments because nanomaterials exhibit size-dependent optical, electrical, and catalytic properties that can be exploited for signal amplification. Second, they allow for miniaturization and portability, leading to the in situ and even real-time detection. Third, their surfaces can be engineered with remarkable precision, enabling selective interactions with target analytes. Finally, nanodevices can be integrated into microfluidic or lab-on-chip platforms, reducing reagent consumption, enhancing throughput, and allowing the development of automated, user-friendly systems [22]. To illustrate the growing importance of this research, Figure 2 presents the annual trend of publications on micro- and nanoplastic research (2014–2023). In the context of MNP detection several categories of nanodevices are particularly relevant. Nanosensors represent one of the most developed areas, harnessing optical, electrochemical, or mechanical transduction mechanisms enhanced by nanomaterials. These devices can detect plastics at extremely low concentrations, sometimes down to the single-particle level, and often within short response times. Optical nanosensors can detect binding events in real time through changes in light absorption or scattering [23]. Electrochemical sensors incorporating nanostructured electrodes can achieve similar results by amplifying redox responses upon particle binding [24]. Lab-on-a-chip platforms, that are microfluidic systems exploit the manipulation of fluids at micrometer scales, work integrating nanostructured materials for selective capture and sensing. The advantage of lab-on-a-chip systems lies in their ability to handle minute sample volumes, automate complex workflows, and achieve high-throughput screening [25]. Nanopore technologies act directly exploiting the passage of particles through nanometer-sized pores. As an MNP translocates through the pore, it produces measurable changes in ionic current, allowing size, shape, and sometimes surface charge to be inferred. This approach, originally developed for biomolecules such as DNA, was adapted to plastic particles and holds particular promise for nanoscale detection [26]. Finally, nanostructured capture materials such as magnetic nanoparticles or functionalized graphene sheets play an essential supporting role. These materials can be engineered to selectively bind or adsorb MNP, enabling pre-concentration from large sample volumes prior to analysis. Such strategies are especially useful in environmental monitoring, where MNP concentrations may be exceedingly low and dispersed across vast volumes of water, soil, or air [27]. All these devices can be designed with built-in capabilities to analyze complex matrixes.

### 3.2. Applications Across Complex Matrices

Environmental, food, and biological samples represent highly complex matrices that pose substantial challenges for the detection and quantification of micro- and nanoplastics (MNPs). These complex matrices can significantly interfere with nanodevice signals by inducing nonspecific adsorption, quenching optical signals, altering surface charges, or promoting aggregation of nanoparticles. Indeed, these matrices contain diverse interfering substances such as dissolved organic matter, salts, colloids, minerals, humic substances, proteins, lipids, and cellular debris [28,29]. Conventional analytical methods typically require extensive pre-treatment—including filtration, digestion, or chemical extraction—to remove such interferences. Magnetic separation and selective polymer functionalization can further enhance matrix tolerance, allowing more reliable and reproducible measurements. However, these steps often risk altering particle properties, introducing artifacts, or causing loss of analytes, thereby limiting reliability, especially at environmentally relevant low concentrations. Nanodevices offer promising solutions to these challenges by integrating functionalized surfaces capable of selectively capturing target polymers while repelling contaminants. This capability reduces non-specific interactions and minimizes the need for labor-intensive sample preparation. For instance, magnetic nanoparticles can be functionalized with polymer-specific ligands, enabling direct capture of polyethylene or polystyrene particles from complex samples. Once bound, an external magnetic field can concentrate and transfer the particles to a detection system for further analysis, such as Raman spectroscopy or fluorescence microscopy [30]. Similarly, microfluidic platforms can integrate multiple separation strategies—such as filtration, size exclusion, electrophoresis, hydrodynamic sorting, or dielectrophoresis—within a single device. Such integration not only streamlines pre-treatment but also allows simultaneous multi-parameter characterization of MNPs, including size, shape, surface charge, and polymer type.

The diversity of sample types—including aquatic systems, soils, sediments, food products, and biological fluids—requires matrix-specific adaptations for nanodevice-based detection.

Aquatic environments, as major sinks and transport pathways for MNPs, present specific challenges such as particle dilution, interference from dissolved organic matter, variable salinity, pH fluctuations, and turbidity [31,32]. These factors hinder the reproducibility and sensitivity of analytical measurements. Nanodevices such as plasmonic optical nanosensors, electrochemical sensors, magnetic nanoparticle-based enrichment systems, and integrated microfluidic separation modules have shown potential for in situ detection of MNPs under laboratory conditions. For example, a recent study used surface-enhanced Raman scattering (SERS) coupled with magnetic nanoparticle separation to detect nanoplastics in seawater samples at sub-micrometer resolution. Other promising approaches include hydrogel-based nanofiltration integrated with portable microfluidic devices for direct monitoring in river and wastewater samples [33].

Soils and sediments, which serve as major reservoirs for MNPs, are characterized by high heterogeneity, abundant minerals, humic substances, and microbial activity. These factors promote particle aggregation and strong adsorption to the matrix, complicating extraction and quantification. Magnetic enrichment using functionalized nanoparticles has been successfully applied to isolate MNPs from sediment cores, enabling downstream characterization by FTIR and electron microscopy [34]. Other approaches include the use of microfluidic sediment fractionation systems that mimic hydrodynamic sorting in natural environments, and polymer-specific affinity membranes that selectively bind microplastic particles. Recent field trials in agricultural soils have demonstrated the feasibility of integrating magnetic enrichment with portable spectroscopic analysis, significantly reducing turnaround time compared to conventional laboratory methods [35].

Food products including seafood, bottled water, honey, salt, and fresh produce—are increasingly recognized as vectors for human exposure to MNPs [36,37]. Contamination arises through packaging, processing, and environmental uptake. Electrochemical nanosensors, optical sensors, and integrated microfluidic digestion–separation platforms have shown capability for direct detection in such complex matrices with minimal preprocessing. For example, a microfluidic digestion device incorporating enzymatic breakdown and size-based separation has enabled detection of nanoplastics in mussel tissue and bottled mineral water with high specificity [38]. Plasmonic nanosensors have been adapted for use in salty matrices such as sea salt and brine solutions, overcoming interference from dissolved salts. Despite these advances, the diversity of food matrices and the absence of standardized regulatory protocols remain major obstacles for widespread implementation [39].

**Figure 3:**
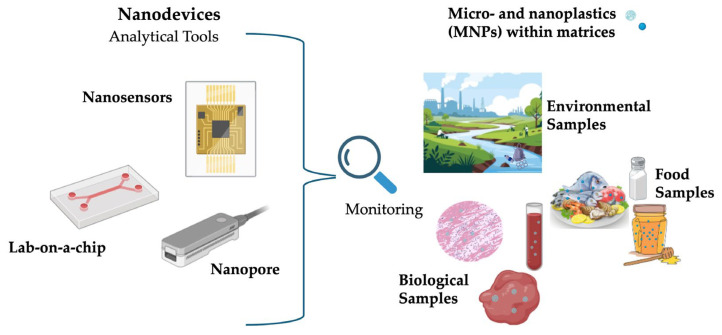
Types of Biological and Environmental Matrices Analyzable by Nanodevices.

Biological matrices—such as human blood, placenta, breast milk, and various tissues—pose particularly formidable challenges. MNP concentrations in biological samples are often extremely low, and detection is complicated by the presence of proteins, lipids, and cellular debris [40,41]. Nanodevices incorporating biofunctionalized sensors have been developed to improve both sensitivity and selectivity. For instance, nanopore-based detection platforms allow real-time identification of particle size and polymer composition directly from biological fluids without extensive preprocessing. Other promising strategies include microfluidic platforms integrating cell lysis, immunocapture, and electrophoretic separation to isolate nanoplastics from plasma or tissue homogenates. Magnetic enrichment coupled with Raman spectroscopy has enabled detection of nanoplastics in human placenta samples, providing valuable insight into exposure pathways and potential health risks [42,43]. Rigorous validation is required, including reproducibility testing, specificity evaluation, inter-laboratory comparisons, regulatory acceptance, and the development of standardized reference materials and cross-platform calibration procedures [44]. Figure 3 schematizes the type of matrices that can be analyzed with nanodevices. Building on the role of nanodevices as functional platforms for MNP monitoring, the next section integrates these concepts within a broader discussion of nanotechnology-based detection strategies, emphasizing how nanoscale material engineering, surface functionalization, and hybrid sensing architectures collectively improve MNP detection.

## 4. Nanotechnology-Based Strategies for MNP Detection

The rapid evolution of nanotechnology lead to new techniques for the detection of micro- and nanoplastics, overcoming many of the intrinsic barriers that have historically limited conventional methods. These strategies build upon the unique physicochemical properties of nanomaterials, their capacity to be engineered with high precision, and the ability to integrate them into compact, multifunctional platforms. Rather than relying solely on bulk spectroscopic signatures or destructive thermal analysis, nanotechnology-based devices can exploit molecular recognition, surface interactions, and nanoscale transduction mechanisms to achieve levels of sensitivity and specificity previously unattainable [45]. Nanotechnology-based strategies for MNP detection can be broadly grouped into four main categories: nanosensors, lab-on-chip platforms, nanopore devices, and nanostructured capture materials. Each of these categories is characterized by a distinctive detection principle and set of advantages, but they are united by their ability to interface with MNP at scales that match or even surpass the dimensions of the particles themselves.

### 4.1. Nanosensors for MNP Detection

Among nanotechnology-enabled strategies, nanosensors represent the most advanced and widely explored class of devices. In the context of micro- and nanoplastic (MNP) detection, nanosensors may exploit optical, electrochemical, or mechanical responses to the presence of plastic particles.

Optical nanosensors are especially promising, because many nanomaterials display size-dependent optical properties that are highly sensitive to local environmental changes. For example, Oh et al. functionalized gold nanoparticles with biomimetic peptides specific to polystyrene nanoplastics and measured localized surface plasmon resonance (LSPR) shifts upon binding, enabling selective detection [46]. The specific recognition of polystyrene (PS) nanoplastics using an oligopeptide probe was achieved through chemical conjugation onto both 40–50 nm gold nanoparticles (AuNPs) immobilized on the LSPR chip and additional 5 nm AuNPs introduced as a sandwiching layer. The peptide probe selectively bound PS nanoplastics prepared as fragmented debris via cryo-grinding. LSPR responses were monitored with a standard UV–Vis spectrophotometer by measuring changes in absorbance and shifts in the plasmonic peak. The sandwich configuration enhanced detection sensitivity by up to 60% owing to consecutive plasmonic coupling effects [46]. Palani et al. demonstrated multispectral LSPR in various Au nanoparticle shapes (spheres, rods) and assessed their sensing performance on microplastics, offering design guidelines for high sensitivity [47]. In particular, they developed a wide-field imaging method, multispectral LSPR (msLSPR), that enables real-time acquisition of full scattering spectra from individual nanoparticles with high spatial, spectral, and temporal resolution. Studies using msLSPR showed that gold nanobipyramids provide more uniform and stronger sensing responses than spheres or rods, as long as their structure remains intact. These findings highlight the critical role of spectral heterogeneity in LSPR sensing and demonstrate the advantages of spectral-domain over intensity-based assays [47]. Optical biosensor reviews further note that SPR/LSPR sensing has already been extended to microplastics (e.g., using estrogen receptors in SPR devices) to detect refractive index perturbations induced by particles, where chromatographic analysis revealed that the surface charge of microplastics largely determined elution time, while estrogen receptors (ERs) provided additional selectivity. Differences observed in SPR sensorgrams suggested a rolling interaction of microplastics on ER surfaces, with quantitative analysis showing a linear relationship between particle number and sensor response units. When ERs were immobilized, binding strength followed the order PS (0.05 nM) > PVC (0.09 nM) > PE (0.14 nM), consistent with stronger and more persistent interactions of polystyrene. Specificity of the ER–microplastic interaction was further confirmed by an ELISA-like magnetic bead assay, and overall results demonstrated the potential of SPR platforms to reveal biologically relevant binding behaviors of plastics [48].

Fluorescent nanomaterials (e.g., quantum dots, carbon dots) act because MNPs may quench fluorescence through energy transfer mechanisms or induce aggregation of the dots, leading to spectral shifts, and in this context an in situ facile synthetic approach was reported to generate carbon quantum dot (CQD) fluorescent markers for polyethylene (PE) using agglomerated silica nanoparticles (SiO_2_) as a support under mild reaction conditions. The resulting PE films, obtained by compression molding, exhibited strong blue fluorescence under UV excitation, attributed to embedded CQDs whose structural and optical properties were confirmed by TEM, UV–vis, and fluorescence spectroscopy. Notably, the emission maximum shifted from 394 to 408 nm when the reaction temperature decreased from 110 to 90 °C, consistent with increased oxygen incorporation, while reactions under inert Ar yielded ultraviolet emission (~286 nm). Such CQD-labeled PE could be easily visualized under 367 nm light, demonstrating its potential as a practical fluorescent tag for polymer identification, traceability in master batches, and improved recycling strategies [49].

Electrochemical nanosensors are composed of nanomaterials such as graphene, carbon nanotubes linked with metallic nanostructures having high surface area and excellent conductivity, which can transduce binding events into electrical signals. For instance, Shabib et al. report that electrode architectures combining nanomaterials and molecular imprinting can achieve detection limits down to ~10^−11^ M for microplastics [50]. Ahmed et al. and others more generally show that modified electrodes can register changes produced by MNP presence via cyclic voltammetry, impedance spectroscopy, or particle-impact strategies [51]. For example, Elli et al. investigated an electrolyte-gated field-effect transistor (EG-FET) sensor employing a carbon nanotube (CNT) semiconducting channel (EG-CNTFET) for nanoparticle detection in aquatic environments. Sensitivity was evaluated using two polystyrene nanoparticle models, one non-functionalized and the other carboxylated, yielding responses of 22.6 μA/(1 mg/mL) and 20.9 μA/(1 mg/mL), respectively. The signal was primarily attributed to hydrophobic interactions between CNTs and the nanoparticles, as further supported by atomic force microscopy visualization of particles adsorbed onto the CNT network. These results demonstrate the feasibility of EG-FET platforms for nanoparticle sensing and provide a foundation for their future application in monitoring environmentally relevant nanoplastics [52].

**Figure 4:**
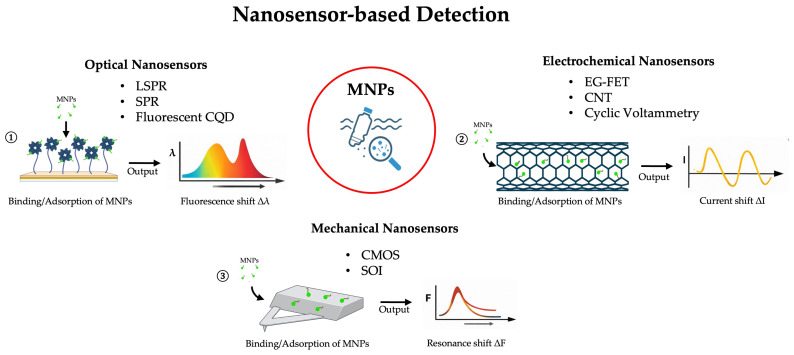
Overview of nanosensor-based technologies for MNP detection, categorized by signal transduction mechanism. ① Optical nanosensors exploit the interaction between light and metallic nanostructures; the binding of an MNP alters the local refractive index, causing a measurable shift in the absorption/resonance spectrum peak. ② Electrochemical nanosensors detect MNPs by measuring variations in electrical properties caused by particle adsorption onto the conductive nanomaterial surface. ③ Mechanical nanosensors function as micro-balances; the added mass of an MNP on the sensor surface induces static deflection or a detectable change in its mechanical resonance frequency.

Mechanical nanosensors, though less frequently applied, show considerable promise. Microcantilever-based devices can detect MNP adsorption via shifts in resonance frequency or cantilever deflection, enabling highly sensitive measurements of particle mass and binding interactions [53]. Recent advances include monolithically integrated microcantilever aptasensors on CMOS chips, which provide enhanced sensitivity and on-chip processing for practical applications. Such a system can consist of a piezoresistive microcantilever array coupled with an on-chip signal processing circuit, enabling compact, high-performance sensing platforms. For example, twelve piezoresistive microcantilevers arranged in three Wheatstone bridge sensors have been fabricated on a single-crystalline silicon device layer of a silicon-on-insulator (SOI) wafer using partially depleted (PD) CMOS technology combined with micromachining processes. This integration exploits the high gauge factor of single-crystalline silicon to achieve low parasitic effects, latch-up, and leakage currents. The device demonstrated a deflection sensitivity of 0.98 × 10^−6^ nm^−1^ and output voltage fluctuations below 1 μV, with the on-chip signal processing circuit achieving a maximum gain of 134.97 and an input offset current of only 0.623 nA. Functionalized with a biotin-avidin system, these sensors detected human IgG, abrin, and staphylococcus enterotoxin B (SEB) with a limit of detection as low as 48 pg/mL, and enabled multichannel detection of SEB, confirming their suitability for high-sensitivity biomolecule detection [54]. Figure 4 and Table 1 recap the examples described.

### 4.2. Lab-on-a-Chip and Microfluidic Platforms

Lab-on-a-chip (LOC) technologies offer a transformative approach to microplastic nanoparticle (MNP) detection by integrating sample preparation, separation, and detection within a single microfluidic device [55,56]. These platforms are particularly attractive for MNP analysis because they can process minute sample volumes, automate complex workflows, and achieve high-throughput screening while minimizing reagent consumption and operator intervention [56,57]. Microfluidic platforms also offer significant advantages beyond serving as simple detection devices, particularly in the context of micro- and nanoplastic (MNP) analysis. Their small-scale, precisely controlled flow environments enable efficient sample preconcentration, size-based fractionation, and matrix cleanup, which are critical for improving the sensitivity and reliability of downstream nanodevice detection. By integrating filtration, dielectrophoretic sorting, or hydrodynamic separation, microfluidics can selectively isolate target particles from complex environmental or biological matrices. This preprocessing capability makes microfluidic devices highly valuable as sample preparation and enrichment tools, complementing sensor technologies and enhancing overall analytical performance.

**Figure 5:**
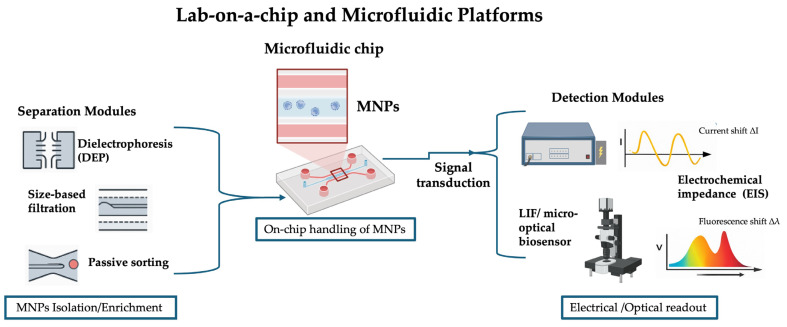
Schematic representation of lab-on-a-chip platforms for micro- and nanoplastic (MNP) analysis. Samples containing MNPs are introduced into the microfluidic chip, where separation and enrichment modules isolate MNPs. The enriched particles are subsequently detected through electrochemical impedance or optical signal transduction, providing quantitative analytical readout.

At the heart of microfluidic platforms lies the ability to precisely control the behavior of fluids at the micrometer scale [58]. This enables techniques such as size-based filtration, hydrodynamic focusing, electrophoresis, and dielectrophoresis, all of which can be adapted to separate MNPs from background matrices [56,59]. Once isolated, the particles can be directed toward integrated detection modules, which often rely on nanosensors or nanostructured surfaces for transduction [60,61]. For example, a polymer-based microfluidic biochip integrating interdigitated electrode arrays (IDAs) was developed to simultaneously separate, manipulate, and detect microparticles through combined dielectrophoresis (DEP) and electrochemical impedance spectroscopy (EIS). The DEP behavior of silica microspheres was systematically characterized, and size-selective separation was demonstrated by sorting microspheres of 1.8 and 3.5 µm diameter into distinct microchambers within a single run. Concurrently, impedance variations induced by microspheres captured on the IDAs enabled quantitative detection. To ensure scalability and affordability, high-throughput polymer microfabrication techniques such as micro-injection molding were employed, facilitating production of low-cost, disposable biochips. This platform thus provides a versatile foundation for multifunctional lab-on-a-chip systems capable of handling and sensing microparticles [62]. Similarly, a laser-induced fluorescence (LIF)-based micro-optical biosensor was developed with enhanced sensitivity and integration. The device combines cyclic olefin copolymer (COC) optical waveguides, a poly(methyl methacrylate) (PMMA) fluidic substrate incorporating an array of microlenses, and a COC coupling prism aligned with the waveguide substrate or cover plate. The fluidic substrate was fabricated by double-sided hot embossing, featuring sampling microchannels on the bottom and microlenses on the top. Waveguides were embedded into the PMMA cover plate by injecting dissolved COC into polydimethylsiloxane (PDMS) lost molds, simultaneously forming the integrated coupling prism. Subsequent fly-cutting reduced the embedded COC waveguides to 50 μm thickness. Thermal fusion bonding of the cover plate and shallow microchannels (aspect ratio 1:20) was achieved using a pressure-assisted boiling point control system, avoiding channel sagging. The large COC prism ensured efficient coupling to the waveguide, with optimal evanescent excitation achieved near the critical angle. The system demonstrated a maximum signal-to-noise ratio (SNR) of 119 and a detection limit of 7.34 × 10^−20^ mol (SNR = 2) for a 100 μm × 50 μm waveguide. The microlens array significantly enhanced fluorescence collection in the sampling zone. This microfabricated waveguide platform enables rapid, low-cost detection of fluorescent MNPs with high sensitivity, low detection limits, and efficient sampling performance [63]. Cristian F. Rodríguez et al. developed a cost-effective passive microfluidic separator for efficient size-based sorting of particles ranging from 15 to 40 µm. The device, fabricated from polymethyl methacrylate (PMMA) substrates via laser ablation, eliminates the need for cleanroom facilities. Its design was optimized through COMSOL 6.4 Multiphysics simulations, and performance testing with chitosan microparticles demonstrated a separation precision of 96.14%. This accessible platform provides laboratories with precise particle control while reducing fabrication costs, offering a practical alternative to traditional cleanroom-based microfluidics [64]. Ahmed A. Elsayed et al. introduced a micro-optofluidic platform designed for rapid quantification of microplastic particles, with simultaneous identification of their chemical composition and size within the 1–100 µm range. The system incorporates micro-reservoirs positioned before micro-filters to concentrate trapped particles in an ultra-compact area, enabling efficient imaging and optical spectroscopy for plastic type determination. In addition, passive size sorting directs particles into reservoirs according to size, while flow cytometry is employed as a complementary reference for size distribution, albeit without chemical specificity. The proof of concept was validated using model samples composed of standard plastic particles of varying sizes and chemical compositions [65]. The described applications are summarized in Figure 5 and Table 2.

### 4.3. Nanopore Technologies

Nanopore-based detection is an approach that capitalizes on nanometer-scale pores to detect individual particles via ionic current fluctuations. Originally developed for DNA sequencing and protein analysis [66], nanopore systems are now being explored for synthetic nanoparticles and, more recently, for MNPs. In a typical nanopore experiment, a single nanopore is embedded in a membrane between two electrolyte chambers. When a voltage is applied, ions flow through the pore generating a baseline current; when an MNP translocates through the pore, it transiently blocks ion flow, producing a detectable current blockade whose amplitude and duration carry information about the size, shape, and surface properties of the particle [67]. This technique offers several compelling advantages for MNP analysis: single-particle resolution enabling direct size distribution measurement rather than averages; label-free operation and real-time detection; and by adjusting pore size or functionalizing pore walls (e.g., with charged or ligand moieties), one can steer selectivity toward certain particle types or surface chemistries.

For example, one study used solid-state nanopores coated with polyimide in a silicon nitride (Si_3_N_4_) membrane to detect 200 nm carboxylated polystyrene nanobeads via resistive pulses, observing that coating length affected signal amplitude and capture dynamics [68]. Another work functionalized thermoplastic polyurethane (TPU) nanopores with polymer brushes (negatively charged poly(acrylic acid), neutral brushes, zwitterionic polymers, etc.) and used tunable resistive pulse sensing (TRPS) to detect 500 nm PS beads; they showed that surface charge had a strong influence on pulse durations and ionic current rectification [69]. Other works use a “needle punching + chemical etching” method to make polymer micro-/nanopores that can then perform resistive pulse detection of nanoparticles. For example, in one study, silver nanoparticles (AgNPs, 56.7 ± 14.1 nm) were aggregated with polystyrene (PS) nanoplastics of 1 µm and 50 nm in the presence of magnesium sulfate (MgSO_4_) as a coagulant. AgNPs were first mixed homogeneously, after which the PS suspension and MgSO_4_ were combined, and 5 µL of the resulting sample was deposited on a silicon wafer and dried. SEM imaging confirmed aggregate formation prior to analysis by SERS, which achieved a lower limit of detection (LOD) than previous work, around 5 µg/mL for both PS particle sizes. Despite interference peaks arising from the spiked river water matrix, characteristic PS signals were clearly identified, demonstrating the sensitivity of SERS for probing nanoplastics [70]. Similarly, Hu et al. [71] developed a quantitative approach using silver nanoparticles (50–60 nm) aggregated with polystyrene nanoplastics through the addition of potassium iodide (KI). In this system, KI acted both as an aggregating agent and as a surface cleaner for AgNPs, improving spectral clarity. PS nanoplastics of different sizes (50, 100, 200, and 500 nm) were successfully detected in aqueous media over a concentration range of 6.25–2000 µg/mL. Reported LODs were 12.5 µg/mL for 50 nm PSNPs, 6.25 µg/mL for 100 nm PSNPs, and 25 µg/mL for both 200 and 500 nm PSNPs, highlighting the potential of this strategy for size-resolved nanoplastics detection.

**Figure 6:**
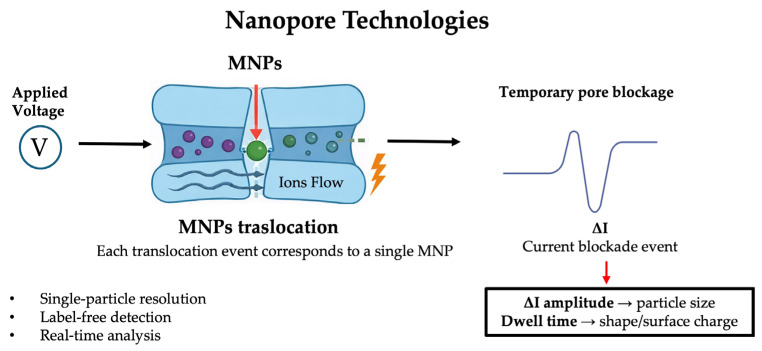
Schematic representation of nanopore-based detection of micro/nanoplastics. Upon application of an external voltage, individual MNPs translocate through the nanopore, inducing transient ionic current blockades. The amplitude and duration of each current pulse provide information on particle size, shape, and surface properties, enabling label-free, single-particle analysis in real time.

Beyond purely resistive detection, advances are pushing toward hybrid readouts: combining resistive pulse sensing with optical (SERS, plasmonic enhancement) signals or integrating nanopores in microfluidic devices to improve stability and handling of samples. For instance, a reusable microfluidic device with a decorated nanopore membrane has been used to detect spherical proteins and gold nanorods, improving ionic conductance and lifetime through surface functionalization and flow control [72]. Figure 6 and Table 3 summarize the described applications.

### 4.4. Nanostructured Capture and Enrichment Materials

Beyond direct sensing technologies, nanotechnology plays a pivotal role in developing advanced materials for capturing, concentrating, and enriching micro- and nanoplastics (MNPs) prior to detection. These materials are especially valuable because environmental and biological concentrations of MNPs are often low, and complex matrices (seawater, soils, food, blood) introduce substantial background interference. Conventional pre-treatment methods such as filtration, digestion, or chemical extraction often risk altering plastic particle properties or losing analytes. Nanostructured capture materials address these limitations through tailored surface chemistries that selectively bind target polymers while repelling contaminants. For example, Fe_3_O_4_@PDA magnetic nanoparticles (magnetite cores coated with polydopamine) have been shown to remove microplastics (MPs) with efficiencies up to ~98.5% in diverse water sources, including rivers, lake water, aquaculture water, and urban water bodies; the PDA shell enhances adhesion via hydrogen-bonding, π-π stacking, and hydrophobic interactions [73]. Similarly, Janus microparticles (MJMs) with asymmetric surfaces constructed using aminated Fe_3_O_4_@SiO_2_ cores achieved ~92% removal efficiency for polystyrene (PS) and ~60% for polyethylene (PE) in water at moderate concentrations (2 mg/mL) within 20 min contact [74]. Graphene-based and other two-dimensional (2D) nanomaterials have also been employed for capture and enrichment. A recent membrane composed of reduced graphene oxide (rGO) nanosheets with Co_3_O_4_ embedded into a polymeric support (h-rGO membranes) demonstrated extremely high removal efficiencies for microspheres and PS beads in wastewater. These membranes have good water flux and environmental stability [75,76]. Additionally, graphene oxide membranes modified with covalent adaptable networks have been used to remove microplastics while maintaining membrane integrity and enabling reusability and closed-loop circularity [77]. Molecularly imprinted polymers (MIPs) are another promising route for selective recognition of plastic types. While many recent MIPs have focused on small molecule pollutants, the concept has been extended toward plastic fragments. For example, some studies embed MIP elements into membranes or composite adsorbents to capture polymer-like molecules [78]. Other hybrid materials also show promise: for instance, tannic-acid-coated Fe_3_O_4_ nanoparticles have been used to recover PS and PET microplastics from water, achieving high removal rates (≈83–98%) under optimal conditions, though performance drops in more complex matrices [79]. All the cited examples are summarized in Table 4.

To facilitate a critical comparison among the diverse nanotechnology-enabled approaches proposed for micro- and nanoplastic (MNP) analysis and mitigation, a consolidated overview of their analytical performance is presented in Table 5. This table summarizes typical orders of magnitude for detection limits, analysis time, sensitivity, cost, and throughput across major technology classes, including nanoplasmonic, electrochemical, microfluidic, nanopore-based, SERS-based, and nanomaterial-assisted removal systems [80,81]. This comparative framework clarifies how different nanotechnological strategies address complementary aspects of MNP detection, quantification, and remediation across environmental and biological matrices.

## 5. Outlook and Conclusions

Nanodevices represent a transformative frontier for the detection of micro- and nanoplastics across complex matrices also if their potential can only be fully realized by addressing several critical needs that span standardization, matrix adaptation, scalability, and traceability. A consistent challenge remains the lack of harmonized definitions, sample preparation protocols, and reporting standards, which significantly hinder the comparability of datasets across studies and the translation of laboratory findings into regulatory practice [16]. Furthermore, matrices such as seawater, sludge, soil, and biological fluids present significant interference from organic matter, salts, and lipids, which necessitates the development of matrix-tailored sensor designs with antifouling capabilities and selective binding properties. Scalability and reproducibility in fabrication, cost-effectiveness, and ease of use are equally important to enable the deployment of nanodevices in routine environmental monitoring. Recent advances have underscored several promising directions, including the development of multi-modal detection platforms that integrate optical, electrochemical, and nanopore technologies to provide richer characterization of particle size, composition, and surface chemistry [82,83]. Emerging materials, such as graphene-oxide membranes and magnetic nanoparticle composites, offer innovative enrichment and capture strategies to enhance sensitivity and selectivity, while microfluidic lab-on-a-chip systems promise integrated pre-treatment, separation, and detection in portable formats suitable for in situ applications. These technological developments must be complemented by robust ethical, regulatory, and safety frameworks, including the establishment of certified reference materials, uniform reporting standards, and interlaboratory validation protocols, to ensure the reliability and comparability of results [84]. Pilot field studies in diverse environmental contexts are essential to evaluate sensor durability, calibration drift, and usability, while engagement with regulatory associations is critical to ensure that emerging nanodevices produce outputs acceptable for policy and risk assessment. By aligning technological innovation with standardization, matrix-specific adaptation, integrated enrichment and detection, and transparent regulatory frameworks, the field can progress toward routine, large-scale monitoring of MNPs. This will not only enable earlier detection at lower size and concentration thresholds but also deepen understanding of the environmental fate and health impacts of plastics, thereby underpinning the development of effective mitigation strategies and informed policy decisions to address plastic pollution before irreversible harm occurs. Moreover, future efforts should prioritize the development of matrix-adapted, scalable, and multimodal nanodevices, coupled with standardized protocols and pilot field validations, to enable reliable, high-throughput monitoring of micro- and nanoplastics and to inform effective environmental mitigation strategies. Emphasis should be placed on integrating sample pretreatment, enrichment, and detection into portable platforms for in situ applications. Collaborative studies with regulatory agencies are needed to establish certified reference materials, uniform reporting standards, and interlaboratory validation, ensuring data comparability and policy relevance. Finally, continued exploration of emerging materials and sensor designs will be critical to push detection limits toward smaller nanoplastic fractions, enabling earlier identification and deeper understanding of environmental fate and health impacts.

## Figures and Tables

**Table 1:** Summary of nanosensor systems for the detection of MNPs across different matrices.

Sensor Type	Nanomaterial Composition/Functionalization	Key Features and Detection Mechanism	Matrix/Application Domain	Challenges and Limitations	Ref.
Plasmonic (LSPR) biosensor	Gold nanoparticles (40–50 nm) functionalized with PS-specific oligopeptide probes; additional 5 nm AuNPs as sandwiching layer	Selective detection of fragmented PS nanoplastics; LSPR peak shift measured via UV–Vis; 60% enhanced sensitivity due to plasmonic coupling	Aquatic environments	Potential interference from coexisting colloids and natural organic matter; limited specificity toward other polymer types; requires precise nanoparticle size control	[46]
Multispectral LSPR (msLSPR)	Gold nanostructures (spheres, rods, bipyramids)	Real-time multispectral imaging; bipyramids showed superior uniformity and stronger responses; provides design guidelines for nanoplasmonic sensing	Biological matrices	Complex data analysis and instrumentation; sensitivity may vary with particle morphology and matrix refractive index fluctuations	[47]
Surface Plasmon Resonance (SPR) biosensor	Estrogen receptor (ER)-functionalized SPR chip	Monitored refractive index perturbations by PS, PVC, and PE; demonstrated bioselective binding and quantification; mimics receptor–plastic interactions	Biological matrices; Aquatics environment; Soil and sediments	Receptor stability and regeneration issues; possible non-specific adsorption; limited throughput for large-scale environmental monitoring	[48]
Fluorescent nanosensor/labeling system	Carbon quantum dots (CQDs) embedded in polyethylene (PE) using SiO_2_ supports	In situ synthesis under mild conditions; strong blue fluorescence (394–408 nm); applicable for polymer identification and traceability	Food products	Applicable mainly to pre-labeled or engineered plastics; limited use for native environmental MNPs; potential photobleaching and matrix fluorescence interference	[49]
Electrochemical nanosensor (MIP-based)	Graphene or carbon nanotube nanocomposites with metallic nanostructures and molecularly imprinted sites	Converts MNP binding into electrical signals; detection limits down to 10^−11^ M; label-free and high surface conductivity	Food products; Aquatic environments	Template removal and imprinting reproducibility challenges; potential fouling in complex matrices; selectivity limited to target imprint	[50]
Electrolyte-Gated FET (EG-CNTFET)	Carbon nanotube semiconducting channel	Sensitive to PS nanoparticles (functionalized and non-functionalized); 22.6 μA and 20.9 μA per mg/mL response; validated by AFM; hydrophobic interactions drive detection	Aquatic environments	Sensitivity strongly dependent on surface chemistry and ionic strength; device-to-device variability; limited polymer discrimination	[52]
Mechanical nanosensor (microcantilever)	CMOS-integrated piezoresistive microcantilever array (12 elements)	On-chip high-sensitivity detection (0.98 × 10^−6^ nm^−1^); <1 μV noise; detected IgG, abrin, SEB (LOD 48 pg/mL); suitable for high-performance biomolecule or MNP sensing	Biological matrices	High fabrication and calibration complexity; sensitivity to temperature and mechanical noise; indirect validation for MNP-specific detection	[54]

**Table 2:** Summary of Lab-on-a-Chip and Microfluidic Platforms for the detection of MNPs across different matrices.

Sensor Type	Nanomaterial Composition/Functionalization	Key Features and Detection Mechanism	Matrix/Application Domain	Challenges and Limitations	Ref.
Microfluidic biochip with interdigitated electrode arrays (IDAs)	Polymer-based microfluidic biochip integrating IDAs for DEP and EIS	Combines dielectrophoresis (DEP) for particle manipulation and electrochemical impedance spectroscopy (EIS) for detection; size-selective separation of 1.8 and 3.5 µm silica microspheres into distinct microchambers; quantitative impedance-based detection; scalable fabrication via micro-injection molding for disposable, low-cost devices	Aquatic environments	Limited discrimination for chemically similar polymers; performance influenced by conductivity and ionic strength of the medium; reduced sensitivity for nanoscale particles	[62]
Laser-induced fluorescence (LIF)-based micro-optical biosensor	Cyclic olefin copolymer (COC) optical waveguides integrated with poly(methyl methacrylate) (PMMA) microfluidic substrate containing microlens array and COC coupling prism	Highly sensitive fluorescence detection platform; hot-embossed PMMA channels and fly-cut COC waveguides (50 μm); SNR = 119, LOD = 7.34 × 10^−20^ mol; microlens array enhances fluorescence collection efficiency; suitable for rapid detection of fluorescently labeled MNPs	Aquatic environments	Requires fluorescent labeling of MNPs; potential interference from background fluorescence; limited applicability to unlabeled or aged environmental plastics	[63]
Passive microfluidic particle separator	Polymethyl methacrylate (PMMA) microchannels fabricated by laser ablation	Passive, cost-effective device for size-based sorting of 15–40 µm particles; optimized by COMSOL Multiphysics; demonstrated 96.14% separation precision with chitosan microparticles; no cleanroom required; accessible and reproducible fabrication	Aquatic environments; Soil and sediments	Restricted to size-based separation; ineffective for sub-micron particles; lacks chemical or polymer-specific identification capability	[64]
Micro-optofluidic platform for microplastic quantification	Micro-reservoir and micro-filter system integrated on a microfluidic chip	Enables simultaneous quantification, size sorting, and spectroscopic identification (1–100 µm range); uses optical spectroscopy for polymer type determination; validated with mixed plastic standards; flow cytometry used as reference for size distribution	Aquatic environments; Soil and sediments	Limited sensitivity below 1 µm; optical signal attenuation in turbid matrices; relatively complex system integration and data processing	[65]

**Table 3:** Summary of Nanopore technologies for the detection of MNPs across different matrices.

Sensor Type	Nanomaterial Composition/Functionalization	Key Features and Detection Mechanism	Matrix/Application Domain	Challenges and Limitations	Ref.
Solid-state nanopore sensor	Polyimide-coated silicon nitride (Si_3_N_4_) nanopores	Detects 200 nm carboxylated PS nanobeads via resistive pulse sensing; coating length influences signal amplitude and capture dynamics; high mechanical stability and reusability	Aquatic environments	Limited throughput due to single-pore operation; signal interpretation sensitive to pore geometry and surface charge; challenges in discriminating mixed polymer types	[68]
Tunable resistive pulse sensor (TRPS)	Thermoplastic polyurethane (TPU) nanopores functionalized with polymer brushes (poly(acrylic acid), neutral, or zwitterionic polymers)	Detects 500 nm PS beads; surface charge strongly affects pulse duration and ionic current rectification; tunable pore properties allow selectivity and control	Aquatic environments; Soil and sediments	Requires careful calibration and mechanical tuning; sensitivity decreases in highly polydisperse samples; fouling and clogging in complex environmental matrices	[69]
SERS-based nanoparticle aggregation platform	Silver nanoparticles (AgNPs, 56.7 ± 14.1 nm) aggregated with PS nanoplastics (1 µm and 50 nm) using MgSO_4_ as coagulant	SEM and SERS used to characterize aggregates; LOD ≈ 5 µg/mL for both PS sizes; clear PS spectral peaks even in spiked river water, confirming strong SERS sensitivity despite matrix interference	Aquatic environments; Soil and sediments	Aggregation-dependent reproducibility; quantitative accuracy affected by variable hotspot formation; limited specificity in mixed polymer systems	[70]
Quantitative AgNP–KI SERS system	Silver nanoparticles (50–60 nm) aggregated with PS nanoplastics via potassium iodide (KI) addition	KI acts as both aggregating and surface-cleaning agent; detects PS nanoplastics of 50–500 nm; LODs: 12.5 µg/mL (50 nm), 6.25 µg/mL (100 nm), 25 µg/mL (200–500 nm); enables size-resolved quantitative detection	Aquatic environments; Soil and sediments	Requires controlled aggregation conditions; sensitivity decreases for irregular or weathered plastics; potential spectral overlap in complex mixtures	[71]
Hybrid nanopore–microfluidic device	Decorated nanopore membrane integrated into reusable microfluidic chip	Detects proteins and gold nanorods; combines resistive and optical sensing; surface functionalization and flow control improve ionic conductance, signal stability, and sensor lifetime	Biological matrices; Food products	Not yet validated for environmental MNPs; fabrication and integration complexity; limited portability for field deployment	[72]

**Table 4:** Summary of Nanostructured capture materials for the detection of MNPs across different matrices.

Sensor Type	Nanomaterial Composition/Functionalization	Key Features and Detection Mechanism	Matrix/Application Domain	Challenges and Limitations	Ref.
Magnetic nanoparticle adsorbent	Fe_3_O_4_@PDA (magnetite nanoparticles coated with polydopamine)	Achieved ~98.5% MP removal efficiency in river, lake, aquaculture, and urban waters; PDA shell enhances adhesion via hydrogen bonding, non covalent (π–π) stacking, and hydrophobic interactions	Aquatic environments	Limited selectivity among different polymer types; potential saturation and reduced efficiency after multiple reuse cycles; possible co-adsorption of natural organic matter	[73]
Janus magnetic microparticles (MJMs)	Aminated Fe_3_O_4_@SiO_2_ cores with asymmetric surface chemistry	Dual-surface functionality enables selective capture; achieved ~92% PS and ~60% PE removal within 20 min at 2 mg/mL; magnetically recoverable and reusable	Aquatic environments	Lower efficiency for non-aromatic polymers (e.g., PE); synthesis complexity; performance sensitive to particle orientation and surface stability	[74]
Graphene-based hybrid membrane	Reduced graphene oxide (rGO) nanosheets embedded with Co_3_O_4_ in a polymeric support (h-rGO)	High water flux, mechanical robustness, and environmental stability; removed PS microspheres efficiently from wastewater; reusable under multiple cycles	Aquatic environments; Soil and sediments	Membrane fouling by organic and inorganic species; limited removal efficiency for nanoplastics below pore-size threshold; fabrication cost and scalability challenges	[75,76]
Covalently adaptable GO membrane	Graphene oxide membrane modified with dynamic covalent adaptable networks	Efficient MP removal with structural integrity retention; reprocessable and recyclable, supporting circular use; suitable for continuous operation	Aquatic environments; Soil and sediments	Long-term chemical stability under harsh water conditions remains uncertain; limited data on polymer-specific selectivity; potential trade-off between adaptability and mechanical strength	[77]
Molecularly imprinted polymer (MIP) composite	Polymer matrix with imprinted cavities for polymer-like structures	High selectivity toward plastic fragments; adaptable for membrane integration or composite adsorbents; demonstrates feasibility for polymer-specific recognition	Aquatic environments; Soil and sediments	Template removal and imprint fidelity challenges; limited binding capacity; decreased performance in heterogeneous environmental samples	[78]
Tannic-acid-coated magnetic nanoparticles	Fe_3_O_4_ nanoparticles coated with tannic acid (polyphenolic surface)	Removed PS and PET MPs with 83–98% efficiency under optimal conditions; magnetic recovery; reduced performance in complex matrices due to fouling	Aquatic environments; Soil and sediments	Susceptibility to surface fouling and competitive adsorption; pH-dependent performance; gradual loss of activity after repeated regeneration cycles	[79]

**Table 5:** Comparative Summary of Nanotechnology-Based Strategies for MNP Detection, Separation, and Removal.

Technology Class	Representative Platforms	Typical LOD	Analysis Time	Sensitivity	Cost	Throughput	Primary Application
**Nanoplasmonic Sensors**	LSPR, msLSPR, SPR biosensors	ng–µg/mL; RIU-based (10^−6^–10^−8^ RIU)	Minutes to <1 h	High (refractive index shifts, bioselective binding)	Moderate–high (optical instrumentation)	Medium (single-point or array-based)	Detection and identification
**Electrochemical and FET-Based Sensors**	MIP-electrochemical sensors, CNT-FET, EG-FET	10^−11^–10^−6^ M; ng/mL	Minutes	Very high (current/impedance modulation)	Moderate	Medium	Detection and quantification
**Mechanical Nanosensors**	Microcantilever arrays	pg–ng/mL (mass-based)	Minutes	Very high (resonance/piezoresistive response)	High (fabrication and control)	Low–medium	High-precision sensing
**Microfluidic and Optofluidic Platforms**	DEP–EIS chips, LIF micro-optical sensors, optofluidic quantifiers	µg/mL to pg/mL (sub-fg for LIF with labeling)	Seconds to minutes	Moderate–high	Low–moderate (disposable chips)	High (continuous flow, parallelization)	Screening, sorting, quantification
**Nanopore-Based Sensors**	Solid-state nanopores, TRPS, hybrid nanopore–microfluidics	Single-particle sensitivity	Seconds to minutes	Very high (event-based detection)	Moderate–high	Low–medium	Size-resolved detection
**SERS-Based Platforms**	AgNP aggregation, KI-assisted SERS systems	~1–25 µg/mL	Minutes	Very high (molecular fingerprinting)	Moderate–high	Low–medium	Polymer identification
**Nanomaterial Capture and Removal Systems**	Magnetic nanoparticles, Janus particles, GO/rGO membranes, MIP composites	Not applicable (removal-focused)	Minutes to hours	High capture efficiency (60–98%)	Low–moderate	High (bulk processing)	Separation and remediation

## Data Availability

No new data were created.

## References

[1] Houssini K., Li J., Tan Q. (2025). Complexities of the global plastics supply chain revealed in a trade-linked material flow analysis. Commun. Earth Environ..

[2] Amobonye A., Bhagwat P., Raveendran S., Singh S., Pillai S. (2021). Environmental Impacts of Microplastics and Nanoplastics: A Current Overview. Front. Microbiol..

[3] Daoutakou M., Kintzios S. (2025). Biosensors for Micro- and Nanoplastics Detection: A Review. Chemosensors.

[4] Harun-Ur-Rashid M., Jahan I., Foyez T., Imran A.B. (2023). Bio-Inspired Nanomaterials for Micro/Nanodevices: A New Era in Biomedical Applications. Micromachines.

[5] Rivera-Rivera D.M., Quintanilla-Villanueva G.E., Luna-Moreno D., Sánchez-Álvarez A., Rodríguez-Delgado J.M., Cedillo-González E.I., Kaushik G., Villarreal-Chiu J.F., Rodríguez-Delgado M.M. (2025). Exploring Innovative Approaches for the Analysis of Micro- and Nanoplastics: Breakthroughs in (Bio)Sensing Techniques. Biosensors.

[6] Berkel C., Özbek O. (2024). Methods used in the identification and quantification of micro(nano)plastics from water environments. S. Afr. J. Chem. Eng..

[7] Pasieczna-Patkowska S., Cichy M., Flieger J. (2025). Application of Fourier Transform Infrared (FTIR) Spectroscopy in Characterization of Green Synthesized Nanoparticles. Molecules.

[8] Rathore C., Saha M., Gupta P., Kumar M., Naik A., Boer J. (2023). Standardization of micro-FTIR methods and applicability for the detection and identification of microplastics in environmental matrices. Sci. Total Environ..

[9] Anger P., von der Esch E., Baumann T., Elsner M., Niessner R., Ivleva N. (2018). Raman Microspectroscopy as a Tool for Microplastic Particle Analysis. TrAC Trends Anal. Chem..

[10] Ou L., Honda A., Miyasaka N., Akaji S., Omori I., Ishikawa R., Li Y., Ueda K., Takano H. (2021). Application of three-dimensional Raman imaging to determination of the relationship between cellular localization of diesel exhaust particles and the toxicity. Toxicol. Mech. Methods.

[11] Liu Y., Hu J., Lin L., Yang B., Huang M., Chang M., Huang X., Dai Z., Sun S., Ren L. (2023). Overcoming the fluorescent interference during Raman spectroscopy detection of microplastics. Sci. Total Environ..

[12] Faltynkova A., Johnsen G., Wagner M. (2021). Hyperspectral imaging as an emerging tool to analyze microplastics: A systematic review and recommendations for future development. Microplastics Nanoplastics.

[13] Silva A.L.P., Silva S.A.M., Duarte A., Barceló D., Rocha-Santos T. (2022). Analytical methodologies used for screening micro(nano)plastics in (eco)toxicity tests. Green Anal. Chem..

[14] Ribeiro F., Duarte A.C., da Costa J.P. (2024). Staining methodologies for microplastics screening. TrAC Trends Anal. Chem..

[15] Groß M., Mail M., Debastiani R., Scherer T., Braun M. (2025). Weathering of plastics in terrestrial environments. TrAC Trends Anal. Chem..

[16] Nene A., Sadeghzade S., Viaroli S., Yang W., Uchenna U.P., Kandwal A., Liu X., Somani P., Galluzzi M. (2025). Recent advances and future technologies in nano-microplastics detection. Environ. Sci. Eur..

[17] Zhu Y., Li Y., Huang J., Zhang Y., Ho Y.-W., Fang J., Lam E. (2024). Advanced Optical Imaging Technologies for Microplastics Identification: Progress and Challenges. Adv. Photonics Res..

[18] Neugirg B., Koebley S., Schniepp H., Fery A. (2016). AFM-based mechanical characterization of single nanofibres. Nanoscale.

[19] Picó Y., Barceló D. (2020). Pyrolysis gas chromatography-mass spectrometry in environmental analysis: Focus on organic matter and microplastics. TrAC Trends Anal. Chem..

[20] La Nasa J., Biale G., Fabbri D., Modugno F. (2020). A review on challenges and developments of analytical pyrolysis and other thermoanalytical techniques for the quali-quantitative determination of microplastics. J. Anal. Appl. Pyrolysis.

[21] Ng H.M., Saidi N.M., Omar F.S., Kasi R., Subramaniam T.R., Bashir S. (2018). Thermogravimetric Analysis of Polymers. Encycl. Polym. Sci. Technol..

[22] Conte R., Foggia R., Valentino A., Di Salle A., Kandsi F., Calarco A. (2024). Nanotechnology advancements transforming molecular diagnostics: Applications in precision healthcare. Int. J. Nano Dimens..

[23] Khani S., Rezaei P. (2024). Optical sensors based on plasmonic nano-structures: A review. Heliyon.

[24] Jalalvand A.R., Karami M.M. (2025). Roles of nanotechnology in electrochemical sensors for medical diagnostic purposes: A review. Sens. Bio-Sens. Res..

[25] Surappa S., Multani P., Parlatan U., Sinawang P.D., Kaifi J., Akin D., Demirci U. (2023). Integrated “lab-on-a-chip” microfluidic systems for isolation, enrichment, and analysis of cancer biomarkers. Lab A Chip.

[26] Cho G., Kim K., Chen W., Son S., Jeon T.-J., Kim S.M. (2024). Nanopore detection of sub-nanosized plastics in PE-coated paper cups and analysis of their inflammatory responses. Chem. Eng. J..

[27] Pervaiz S., Javed M., Shah A., Latif A., Nasir S., Shah I. (2025). Environmental applications of magnetic nanohybrid materials. RSC Adv..

[28] Tao S., Lin B. (2000). Water soluble organic carbon and its measurement in soil and sediment. Water Res..

[29] Chiu M.L., Lawi W., Snyder S.T., Wong P.K., Liao J.C., Gau V. (2010). Matrix Effects—A Challenge toward Automation of Molecular Analysis. SLAS Technol..

[30] Al-Amiery A.A., Fayad M.A., Abdul Wahhab H.A., Al-Azzawi W.K., Mohammed J.K., Majdi H.S. (2024). Interfacial Engineering for Advanced Functional Materials: Surfaces, Interfaces, and Applications. Results Eng..

[31] Koelmans A.A., Mohamed Nor N.H., Hermsen E., Kooi M., Mintenig S.M., De France J. (2019). Microplastics in freshwaters and drinking water: Critical review and assessment of data quality. Water Res..

[32] Gigault J., Halle A.T., Baudrimont M., Pascal P.Y., Gauffre F., Phi T.L., El Hadri H., Grassl B., Reynaud S. (2018). Current opinion: What is a nanoplastic?. Environ. Pollut..

[33] Asamoah B.O., Uurasjärvi E., Räty J., Koistinen A., Roussey M., Peiponen K.-E. (2021). Towards the Development of Portable and In Situ Optical Devices for Detection of Micro-and Nanoplastics in Water: A Review on the Current Status. Polymers.

[34] Rillig M.C., Ingraffia R., de Souza Machado A.A. (2017). Microplastic Incorporation into Soil in Agroecosystems. Front. Plant Sci..

[35] Zhu X., Wang K., Yan H., Liu C., Zhu X., Chen B. (2022). Microfluidics as an Emerging Platform for Exploring Soil Environmental Processes: A Critical Review. Environ. Sci. Technol..

[36] Smith M., Love D.C., Rochman C.M., Neff R.A. (2018). Microplastics in Seafood and the Implications for Human Health. Curr. Environ. Health Rep..

[37] Kumar V., Sharma N., Umesh M., Gupta P., Sharma P., Basheer T., Huligowda L.K.D., Thomas J., Bhagat S.K., Pasrija R. (2024). Microplastics in food: Occurrence, toxicity, green analytical detection methods and future challenges. Green Anal. Chem..

[38] Hassan M.M., Yi X., Zareef M., Li H., Chen Q. (2023). Recent advancements of optical, electrochemical, and photoelectrochemical transducer-based microfluidic devices for pesticide and mycotoxins in food and water. Trends Food Sci. Technol..

[39] Singh R., Dutt S., Sharma P., Sundramoorthy A.K., Dubey A., Singh A., Arya S. (2023). Future of Nanotechnology in Food Industry: Challenges in Processing, Packaging, and Food Safety. Challenges.

[40] Leslie H.A., van Velzen M.J.M., Brandsma S.H., Vethaak A.D., Garcia-Vallejo J.J., Lamoree M.H. (2022). Discovery and quantification of plastic particle pollution in human blood. Environ. Int..

[41] Ragusa A., Svelato A., Santacroce C., Catalano P., Notarstefano V., Carnevali O., Papa F., Rongioletti M.C.A., Baiocco F., Draghi S. (2021). Plasticenta: First evidence of microplastics in human placenta. Environ. Int..

[42] Banigo A.T., Azeez T.O., Ejeta K.O., Lateef A., Ajuogu E. (2020). Nanobiosensors: Applications in biomedical technology. IOP Conf. Ser. Mater. Sci. Eng..

[43] Kumar P., Lata K., Gacem A., Tariq M., Singh S., Sharma A., Yadav V.K., Bhutto J.K., Kumar M., Alreshidi M.A. (2025). A review on the environmental fate, toxicological risks, and cutting-edge degradation methods of microplastics contamination. Environ. Sci. Eur..

[44] Boctor J., Hoyle F.C., Farag M.A., Ebaid M., Walsh T., Whiteley A.S., Murphy D.V. (2025). Microplastics and nanoplastics: Fate, transport, and governance from agricultural soil to food webs and humans. Environ. Sci. Eur..

[45] Darwish M.A., Abd-Elaziem W., Elsheikh A., Zayed A.A. (2024). Advancements in nanomaterials for nanosensors: A comprehensive review. Nanoscale Adv..

[46] Oh S., Hur H., Kim Y., Shin S., Woo H., Choi J., Lee H.H. (2021). Peptide Specific Nanoplastic Detection Based on Sandwich Typed Localized Surface Plasmon Resonance. Nanomaterial.

[47] Palani S., Kenison J.P., Sabuncu S., Huang T., Civitci F., Esener S., Nan X. (2023). Multispectral Localized Surface Plasmon Resonance (msLSPR) Reveals and Overcomes Spectral and Sensing Heterogeneities of Single Gold Nanoparticles. ACS Nano.

[48] Huang C.J., Narasimha G.V., Chen Y.C., Chen J.K., Dong G.C. (2021). Measurement of Low Concentration of Micro-Plastics by Detection of Bioaffinity-Induced Particle Retention Using Surface Plasmon Resonance Biosensors. Biosensors.

[49] Yin S., Duvigneau J., Vancso G.J. (2021). Fluorescent Polyethylene by In Situ Facile Synthesis of Carbon Quantum Dots Facilitated by Silica Nanoparticle Agglomerates. ACS Appl. Polym. Mater..

[50] Shabib A., Maraqa M.A., Mohammad A.F., Awwad F. (2025). Design, fabrication, and application of electrochemical sensors for microplastic detection: A state-of-the-art review and future perspectives. Environ. Sci. Eur..

[51] Lohith Kumar D.H., Bhardwaj G., Indhur R., Wankhede L., Brar S.K., Kumari S. (2025). Electrochemical approaches for detecting micro and nano-plastics in different environmental matrices. Int. J. Electrochem. Sci..

[52] Elli G., Ciocca M., Shkodra B., Ibba P., Lugli P., Petti L. Electrolyte-Gated Field-Effect Transistor-Based Sensor for Nanoplastic Detection: A Sensitivity Investigation of Two Nanoplastic Models. Proceedings of the 2024 IEEE Sensors.

[53] Setiono A., Bertke M., Nyang’au W.O., Xu J., Fahrbach M., Kirsch I., Uhde E., Deutschinger A., Fantner E.J., Schwalb C.H. (2020). In-Plane and Out-of-Plane MEMS Piezoresistive Cantilever Sensors for Nanoparticle Mass Detection. Sensors.

[54] Liu Y., Tian Y., Lin C., Miao J., Yu X. (2023). A monolithically integrated microcantilever biosensor based on partially depleted SOI CMOS technology. Microsyst. Nanoeng..

[55] Kumari M., Gupta V., Kumar N., Arun R.K. (2024). Microfluidics-Based Nanobiosensors for Healthcare Monitoring. Mol. Biotechnol..

[56] Wang W., Xia L., Xiao X., Li G. (2024). Recent Progress on Microfluidics Integrated with Fiber-Optic Sensors for On-Site Detection. Sensors.

[57] Lan Z., Chen R., Zou D., Zhao C.-X. (2025). Microfluidic Nanoparticle Separation for Precision Medicine. Adv. Sci..

[58] Ardila C.M., Jiménez-Arbeláez G.A., Vivares-Builes A.M. (2023). The Potential Clinical Applications of a Microfluidic Lab-on-a-Chip for the Identification and Antibiotic Susceptibility Testing of Enterococcus faecalis-Associated Endodontic Infections: A Systematic Review. Dent. J..

[59] Wu K., He X., Wang J., Pan T., He R., Kong F., Cao Z., Ju F., Huang Z., Nie L. (2022). Recent progress of microfluidic chips in immunoassay. Front. Bioeng. Biotechnol..

[60] Venugopalan P., Samad S.A., Kumawat N., Kumar S. (2025). Plasmonic sensing in microfluidic paper-based analytical devices integrated with metal nanoparticles: A review. RSC Adv..

[61] Jiang W., Ma Z., Cao F., Hu L., Bao L., Chang P., Xu C., Lv X., Xie Y. (2023). Label-free integrated microfluidic plasmonic biosensor from vertical-cavity surface-emitting lasers for SARS-CoV-2 receptor binding domain protein detection. Opt. Express.

[62] Zou Z., Lee S., Ahn C. (2008). A Polymer Microfluidic Chip with Interdigitated Electrodes Arrays for Simultaneous Dielectrophoretic Manipulation and Impedimetric Detection of Microparticles. Sens. J. IEEE.

[63] Park D.S., Young B.M., You B.H., Singh V., Soper S.A., Murphy M.C. (2020). An integrated, optofluidic system with aligned optical waveguides, microlenses, and coupling prisms for fluorescence sensing. J. Microelectromechanical Syst..

[64] Rodríguez C.F., Guzmán-Sastoque P., Gantiva-Diaz M., Gómez S.C., Quezada V., Muñoz-Camargo C., Osma J.F., Reyes L.H., Cruz J.C. (2023). Low-cost inertial microfluidic device for microparticle separation: A laser-Ablated PMMA lab-on-a-chip approach without a cleanroom. HardwareX.

[65] Elsayed A.A., Erfan M., Sabry Y.M., Dris R., Gaspéri J., Barbier J.-S., Marty F., Bouanis F., Luo S., Nguyen B.T.T. (2021). A microfluidic chip enables fast analysis of water microplastics by optical spectroscopy. Sci. Rep..

[66] He H., Scheicher R.H., Pandey R., Rocha A.R., Sanvito S., Grigoriev A., Ahuja R., Karna S.P. (2008). Functionalized Nanopore-Embedded Electrodes for Rapid DNA Sequencing. J. Phys. Chem. C.

[67] Fried J.P., Swett J.L., Nadappuram B.P., Fedosyuk A., Sousa P.M., Briggs D.P., Ivanov A.P., Edel J.B., Mol J.A., Yates J.R. (2021). Understanding Electrical Conduction and Nanopore Formation During Controlled Breakdown. Small.

[68] Leong I.W., Tsutsui M., Nakada T., Taniguchi M., Washio T., Kawai T. (2019). Back-Side Polymer-Coated Solid-State Nanopore Sensors. ACS Omega.

[69] Srinivas A.R.G., Hilali R., Damavandi M., Malmstrom J., Barker D., Weatherall E., Willmott G., Travas-Sejdic J. (2021). Polymer Brush Functionalization of Polyurethane Tunable Nanopores for Resistive Pulse Sensing. ACS Appl. Polym. Mater..

[70] Zhou X.-X., Liu R., Hao L.-T., Liu J.-F. (2021). Identification of polystyrene nanoplastics using surface enhanced Raman spectroscopy. Talanta.

[71] Hu R., Zhang K., Wang W., Wei L., Lai Y. (2022). Quantitative and sensitive analysis of polystyrene nanoplastics down to 50 nm by surface-enhanced Raman spectroscopy in water. J. Hazard. Mater..

[72] Roman J., Jarroux N., Patriarche G., Français O., Pelta J., Le Pioufle B., Bacri L. (2017). Functionalized Solid-State Nanopore Integrated in a Reusable Microfluidic Device for a Better Stability and Nanoparticle Detection. ACS Appl. Mater. Interfaces.

[73] Li Y., Chen H., Li S., Feng L., Wang Z., Wang D., Wang Q., Wang H. (2024). Corals-inspired magnetic absorbents for fast and efficient removal of microplastics in various water sources. RSC Adv..

[74] Li W., Liu S., Huang K., Qin S., Liang B., Wang J. (2023). Preparation of magnetic Janus microparticles for the rapid removal of microplastics from water. Sci. Total Environ..

[75] Khan S., Kalsoom U., Kashif M., Hussain S.A., Gul M., Azizi S., Maaza M. (2025). Smart and Sustainable Microplastic Removal: Hybrid Systems, Bio-Inspired Technologies, Real-Time Sensing, and Policy Integration. Water Air Soil Pollut..

[76] Zhang W., Xu H., Xie F., Ma X., Niu B., Chen M., Zhang H., Zhang Y., Long D. (2022). General synthesis of ultrafine metal oxide/reduced graphene oxide nanocomposites for ultrahigh-flux nanofiltration membrane. Nat. Commun..

[77] Sen Gupta R., Mandal S., Malakar A., Rege S., Islam S.S., Samanta K., Misra A., Bose S. (2024). Graphene oxide offers precise molecular sieving, structural integrity, microplastic removal, and closed-loop circularity in water-remediating membranes through a covalent adaptable network. J. Mater. Chem. A.

[78] Enyoh C.E., Devi A., Maduka T.O., Tyagi L., Rana S., Akuwudike I.S., Wang Q. (2025). A Review of Materials for the Removal of Micro- and Nanoplastics from Different Environments. Micro.

[79] Sacko A., Nure J.F., Nyoni H., Mamba B., Nkambule T., Msagati T.A.M. (2024). The Application of Tannic Acid-Coated Magnetite Nanoparticles for Recovery of Microplastics from the Water System. Water Conserv. Sci. Eng..

[80] Sujathan S., El-Zein A. (2026). Performance of analytical techniques for microplastic and nanoplastic quantification in the presence of clay. Water Res..

[81] Magalhães S., Alves L., Medronho B., Svanedal I., Norgren M., Rasteiro M. (2025). Innovative Approaches to Mitigating Microplastic Pollution in Effluents and Soils. Sustainability.

[82] Choi S., Lee S., Kim M.-K., Yu E.-S., Ryu Y.-S. (2024). Challenges and Recent Analytical Advances in Micro/Nanoplastic Detection. Anal. Chem..

[83] Zhang H., Duan Q., Yan P., Lee J., Wu W., Zhou C., Zhai B., Yang X. (2025). Advancements and challenges in microplastic detection and risk assessment: Integrating AI and standardized methods. Mar. Pollut. Bull..

[84] Kamel A.H., Hefnawy A., Hazeem L.J., Rashdan S.A., Abd-Rabboh H.S.M. (2024). Current perspectives, challenges, and future directions in the electrochemical detection of microplastics. RSC Adv..

